# Cystatin C-Based Equations Detect Hidden Kidney Disease and Poor Prognosis in Newly Diagnosed Patients with Multiple Myeloma

**DOI:** 10.1155/2022/4282226

**Published:** 2022-04-16

**Authors:** Francisco-Javier Cepeda-Piorno, Esther González-García, Alba Méndez-Gallego, Juan Torres-Varona, Vanesa García-Moreira, Christian Sordo-Bahamonde, Cristina AlberdiGarcía-del-Castillo, Elene Astobieta-Madariaga, Maria-Victoria Mateos-Manteca, Segundo González-Rodríguez

**Affiliations:** ^1^Servicio Análisis Clínicos, Hospital Universitario de Cabueñes, Instituto de Investigación Biosanitaria Del Principado de Asturias (ISPA), Gijón, Spain; ^2^Servicio de Hematología, Hospital Universitario de Cabueñes, ISPA, Gijón, Spain; ^3^Hospital Universitario de Cabueñes, Gijón, Spain; ^4^Hospital Universitario de Cabueñes, Servicio de Hematología, Gijón, Spain; ^5^Servicio de Análisis Clínicos, Hospital Universitario de Cabueñes, Gijón, Spain; ^6^ISPA, Universidad de Oviedo, Oviedo, Spain; ^7^Servicio de Análisis Clínicos, Hospital Universitario Cabueñes, Gijón, Spain; ^8^Hospital Universitario de Salamanca, Servicio de Hematología, Salamanca, Spain

## Abstract

**Objectives:**

The aim of this study was to compare the creatinine equations with cystatin C (CysC) equations to define renal impairment (RI) in newly diagnosed multiple myeloma (MM) patients and to analyse the equation that allows for identifying patients with more and worse prognostic factors.

**Methods:**

Renal function was evaluated prospectively in 61 patients with newly diagnosed untreated MM employing CKD-EPI and CAPA equations. The comparison was conducted using Bland–Altman graphics and Cohen's Kappa statistic. Mann–Whitney *T* and Chi-square tests were used, and univariate and multivariate analyses were carried out.

**Results:**

According to the IMWG criteria, 26% of patients showed RI (3 women/13 men) whilst the use of CysC equations allowed us to identify up to 39% of patients (7 women/17 men). The CAPA equation was less biased and dispersed and more sensitive than CKD-EPI-creatinine. Furthermore, univariate analysis unveiled an association between decreased CKD-EPI-CysC and poor prognosis based on R-ISS-3.

**Conclusions:**

The IMWG criteria may underestimate kidney disease, mostly in women, which could affect the dose received as well as its toxicity. Altogether, our data suggest that equations that include CysC are more accurate to detect hidden kidney disease, as well as patients with more and worse prognostic factors, in newly diagnosed MM.

## 1. Introduction

Multiple myeloma (MM) is characterized by the clonal expansion of malignant plasma cells in bone marrow. MM is the second most common hematological malignancy, representing approximately 10% of cases and 1% of all cancer diagnoses [1]. With a median age at diagnosis of 65 years, the annual incidence is about 3–5 cases per 100,000 people [2]. Diagnosis of MM is performed according to the International Myeloma Working Group (IMWG) criteria that allows the classification of this multistep evolutionary disease from an early asymptomatic stage, known as monoclonal gammopathy of undetermined significance (MGUS), an intermediate smoldering stage (sMM), and symptomatic MM [3, 4].

Despite novel therapeutic agents, including immunomodulatory drugs, small molecule inhibitors, or monoclonal antibodies, being revolutionized the landscape of MM therapy, it still remains as an incurable disease [5]. However, only patients with myeloma-related symptoms, such as anemia, hypercalcemia, bone disease, or renal impairment (RI), are considered for treatment initiation [2, 4]. In addition, those patients owning biomarkers predicting high risk of progression (>80%) to myeloma-defining events (MDE) are also considered for therapy [3]. These MDE include, among others, involved/uninvolved light chain index higher than 100, two or more focal lesions, and bone marrow infiltration by plasma cells ≥60%.

Importantly, MDE include RI and it is considered as a poor prognosis factor. RI is a common complication in patients with MM and correlates with diminished time to treatment and overall survival [6]. In this context, the accurate identification of kidney disease is crucial, since recovery of RI is associated with response to therapy [6]. For defining RI (IMGW), serum creatinine (sCr) (>2 mg/dL) or creatinine clearance (CrCl) (<40 mL/min) is employed, although both parameters are considered to underestimate RI since sCr may vary depending on age or muscle mass [7–9]. Renal function is usually estimated using sCr using Chronic Kidney Disease Epidemiology Collaboration (CKD-EPI)-based equation in order to estimate glomerular filtration rate (eGFR). Nevertheless, international guidelines recognize that equations based on sCr are imprecise and they do not represent the most accurate method for evaluating RI, especially in elderly patients owning malnutrition and fragility, very common characteristics of patients with MM [10]. In this context, the Kidney Disease Improving Global Outcomes (KDIGO) recommends equations based on the combination of sCr and cystatin C (CysC) (CKD-EPI-sCr-CysC) to estimate GFR for chronic kidney disease or RI in patients under treatment using drugs with narrow therapeutic range [11]. There are new equations that include CysC and could provide advantages (like CAPA equation (Caucasian and Asian pediatric and adult subjects)), but their validation is required prior to their widespread use in clinical practice.

The aim of this study was to compare different equations with sCr and CysC to define RI according to IMWG criteria and to know the most sensitive equation in detecting risk patients in newly diagnosed and untreated MM.

## 2. Materials and Methods

### 2.1. Patients

61 consecutive newly diagnosed and untreated patients with MM (24 female and 37 male) were enrolled in the study between December 2018 and April 2021. This epidemiologic study was approved by the local ethics committee and the Research Committee from Hospital Universitario de Cabueñes (Spain). MM was diagnosed according to standard clinical and laboratory criteria established by international guidelines [3]. Clinical and laboratory data were collected from hospital medical records. All patients signed the informed consent to participate in the study.

sCr measures were performed following a method that can be traced to an IDMS (isotope dilution mass spectrometry) reference procedure, picrate-based method (Advia 2400, Siemens). CysC values were detected employing a nephelometric assay traceable to the international calibrator (Dimension Vista, Siemens). Serum and urine monoclonal components were determined by capillary electrophoresis (Capilarys 2, Sebia). Turbidimetric measurement of levels of serum free light chains was performed following the Freelite assay (SPA-Plus, Binding-Site).

### 2.2. Statistics

We used the CKD-EPI equations according to KDIGO guidelines, and CKD-EPI-sCr-CysC has been considered as a “gold-standard,” given the unavailability of alternative measurements, such as inulin or Cr-EDTA, in the clinic, according to these guidelines. The CAPA equation was defined by Grubbs et al. as follows:

(1) $$\begin{array}{c} 130\times {\text{CysC}}^{-1.069}\times {\text{age}}^{-0.117}-7. \end{array}$$

For the data analysis, the values corresponding to the continuous variables have been depicted as mean, median, 95% CI, or percentage, depending on the variable. The comparisons between the different equations were made with Bland–Altman graphics, and to compare the classification in chronic kidney disease stages, we employed the Kappa statistic value. Likewise, the comparison between the distinct parameters associated with eGFR reduction determined with sCr or CysC was evaluated using Student's *t*-test (normal distribution) or Mann–Whitney *U* test (nonparametric distribution) for the quantitative variables and Chi-square or Fisher's exact test for qualitative variables. *P* values lower than 0.05 were considered as statistically significant. To determine the correlation between CKD-EPI reduction (with CysC or sCr) considering poor prognosis factors in patients with MM, multivariate analyses were performed, including all the relevant clinical variables based on the prior univariate analysis. All statistical analyses were performed using Med.Calc software (version 9.2.1.0) and SPSS (version 24).

## 3. Results

**Figure 1:**
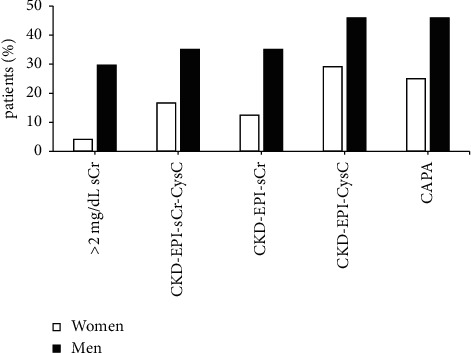
Newly diagnosed multiple myeloma patients with renal impairment, according to international myeloma working group criteria (categorized by sex) using serum creatinine and cystatin C in different equations.

The characteristics of the patients included in the study are shown in Table 1. Renal function was evaluated in a cohort of 61 patients with newly diagnosed MM using different equations that include sCr and/or CysC values (Figure 1). Based on IMWG criteria, 12 patients (19.6%) had sCr levels higher than 2 mg/dL and 17 of them (27.8%) showed CKD-EPI-sCr-CysC lower than 40 mL/min/1.73 m^2^. In contrast, the CKD-EPI-sCr equation rendered 16 patients (26.2%) with RI, whereas 24 (39.3%) and 23 (37.7%) patients were detected using CKD-EPI-CysC and CAPA equations, respectively (Table 2). Thus, those equations including CysC estimated a higher number of patients with RI that those equations considering only sCr values. These differences were more pronounced in women (12% with CKD-EPI-sCr vs. 29% and 25% with CKD-EPI-CysC and CAPA, respectively). Overall, the performance of the different equations evaluated to define RI according to IMWG criteria was very good for CKD-EPI-sCr (Kappa index = 0.958 (0.88–1, 95% CI) and good for CKD-EPI-CysC and CAPA ((Kappa index = 0.747 (0.577–0.917, 95% CI) and Kappa index = 0.779 (0.619–0.939, 95% CI), respectively)).

**Figure 2:**
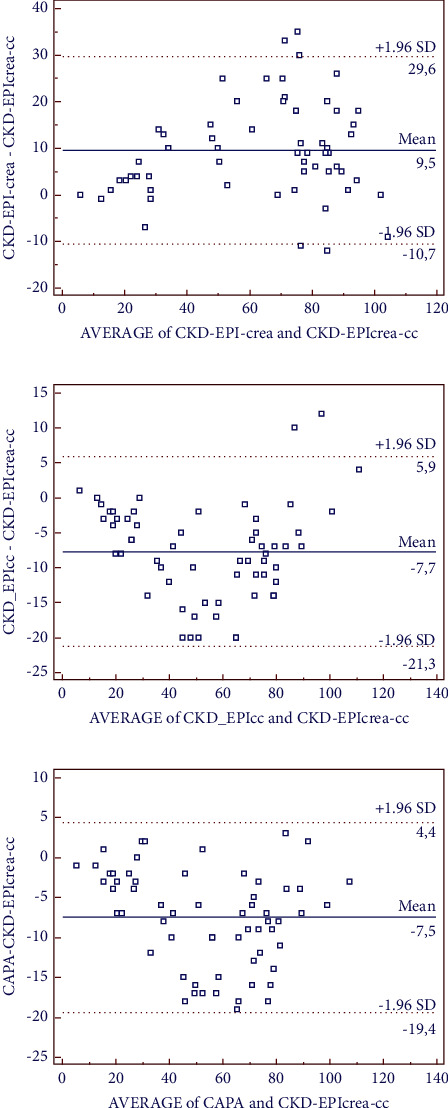
Comparison of CKD-EPI-creatinine, CKD-EPI-cystatin C, and CAPA equations with CKD-EPI-crea-CysC equation (using with reference method according to KDIGO guidelines) in newly diagnosed multiple myeloma patients.

CKD-EPI-sCr was also less sensitive than the equations including CysC in the detection of patients with chronic kidney disease (stage 3), identifying 21 patients vs. 35 patients with the preferred equations (Table 2). The equations that include CysC values estimated the same number of patients with chronic kidney disease (Kappa index = 1). In addition, these equations were more sensitive in detecting eGFR <60 mL/min/1.73 m^2^, corresponding to chronic kidney disease stage 3, compared to CKD-EPI-sCr. Particularly, the CAPA equation was less biased (−7.5 mL/min/1.73 m^2^) and dispersed (−19.4 to 4.4 mL/min/1.73 m^2^, 95% CI), while CKD-EPI-sCr showed the highest bias (+9.5 mL/min/1.73 m^2^) and imprecision (−10.7 to 29.6 mL/min/1.73 m^2^) (Figure 2).

In addition, we compared the characteristics of patients with MM suffering from kidney disease (eGFR <60 mL/min/1.73 m^2^) estimated by CKD-EPI-sCr and by CKD-EPI-CysC, unveiling similar profiles in these individuals (Table 3). Compared to patients with normal eGFR, the cohort of patients with RI estimated using both equations was older and displayed significantly higher *β*-2-microglobulin, serum urate, proteinuria, and serum monoclonal component levels and lower values of hemoglobin. Moreover, the majority of these patients showed advanced MM (R-ISS stage 3). Of note, patients with chronic kidney disease estimated with CKD-EPI-CysC had lower levels of albumin (34.1 g/L versus 38.05 g/L; *P*=0.0137) (Table 3), a risk factor associated to MM. Fourteen patients with CKD-EPI-CysC reduction, but no CKD-EPI-sCr, have distinctively low albumin levels as well (32 g/L). No significant differences were observed in the rest of variables analyzed. Among these high-risk patients, three died within ten months from diagnosis (2 men and 1 woman) due to COVID-19, sudden death, and sepsis (CKD-EPI-CysC 35, 41, and 25 mL/min/1.73 m^2^ versus CKD-EPI-sCr 88, 83, and 64 mL/min/1.73 m^2^, respectively).

Finally, univariate analysis unraveled a significant association between reduced eGFR levels estimated by CKD-EPI-CysC and poor prognosis of patients with newly diagnosed MM based on R-ISS-3 (HR = 14.73; range 1.785–122.23, 95% CI, *P*=0.013). Multivariate analysis including age, sex, *β*-2-microglobulin, hemoglobin, albumin, and proteinuria as variables, further showed that CKD-EPI-CysC reduction was the only independent prognostic factor for poor prognosis indicated by the R-ISS-3 staging score.

## 4. Discussion

Renal disease is a common complication associated with poor prognosis in MM. sCr and CrCl are classically used to detect eGFR decline, but they may underestimate their prevalence, particularly in patients with MM [12]. Measurement of GFR using inulin infusion or radiotracers is one of the most accurate methods to evaluate the renal function, but it is time-consuming, expensive, and not available in most hospitals [5]. Alternatively, eGFR CysC-based equations are more sensitive, cheaper, and easy to implement [12–14]. The FDA (Food and Drug Administration) and EMA (European Medicines Agency) recommend these equations for drug dose adjustment and for renal disease evaluation [15]. Despite their clinical impact, there are not clear indications about which equation must be used [15]. The criteria for diagnosis of RI may have a significant impact in the use of nephrotoxic drugs, such as some antibiotics, chemotherapeutic drugs, or nonsteroidal anti-inflammatory drugs (NSAIDs), the adjustment of the dose of bisphosphonates, or the use of intravenous contrasts, which may quickly impair renal disease in undiagnosed patients with MM.

In this study, we compare different equations based on CysC (CKD-EPI-CysC and CAPA) and sCr (CKD-EPI-sCr) to estimate the occurrence of renal disease in 61 newly diagnosed patients with MM. Our findings show that the equations based on CysC are more sensitive than sCr, duplicating the number of patients with renal disease detected (39% vs. 19%). The equations based on CysC were also more sensitive in detecting chronic renal disease (stage 3) than CKD-EPI-sCr (35 vs. 28 patients detected). Of note, the CAPA equation showed the least bias and imprecision compared to the reference method [16]. This equation uses an international calibrator, it is easy to implement (it only requires the age and CysC levels of the patient without race or sex), and our study is the first that shows a 100% concordance with CKD-EPI-CysC (eGFR <60 mL/min/1.73 m^2^) in newly diagnosed MM patients. Furthermore, our study brings to light that sCr-based methods are particularly inaccurate detecting renal disease in female patients. Using sCr, we only detected 1 woman with renal disease, whereas CKD-EPI-sCr (<40 mL/min/1.73 m^2^), the most frequently used equation in most hospitals, detected 3 women. Contrasting these data, 7 female patients with RI were identified using the CKD-EPI-CysC equation. sCr levels highly depend on the muscle mass, and thus, it might not be adequate to use the same cutoff for both male and female patients. The fact that CysC values are not affected by muscle mass, sex, or age may explain the higher accuracy of CysC compared to sCr to diagnose renal disease, particularly, in female patients. In addition, Jaffe's method, despite not being specific to sCr, is still the most widely employed to determine this value. Nevertheless, Jaffe's reaction may also occur with other chromogenic substances (such as cephalosporins or bilirubin), and monoclonal proteins may also interfere with this analysis in patients with MM [17–19]. In contrast, CysC analysis may be altered by thyroid disease or high-dose corticosteroid treatment, a scenario we cannot evaluate in this study, since none of the newly diagnosed patients included had these features.

Equations based on CysC may be useful for an early detection of patients with renal disease. In MM, eGFR <40 ml/min/1.73 m^2^ has been established for RI, but eGFR <60 mL/min/1.73 m^2^ for more than 3 months is used for the general population, because it detects patients with increased risk of mortality in a more accurate way [11]. In this study, we used a stricter cutoff (60 mL/min/1.73 m^2^) for the analysis of patients with MM. This allowed the detection of a group of patients with MM with low level of serum albumin, which is well known to be associated with poor nutritional status and higher liver alteration. It may also reflect higher inflammation, since serum albumin has been associated with interleukin 6 (IL-6), a potent inflammatory mediator and promoter of myeloma cell growth. Likewise, it has been associated with poor prognosis of patients with MM [20]. Furthermore, there is also increasing evidence that CysC may be associated with bone disease in MM, and it has been proposed as a biomarker and, further, as a potential therapeutic target in this type of cancer [13, 21]. CysC has also been shown to identify a subset of ISS-3 score patients with worse outcome [13]. In agreement, univariate and multivariate analyses showed that worse outcome for CKD-EPI-CysC reduction (<60 mL/min/1.73 m^2^) is an independent prognosis factor according to the R-ISS-3 score in our study.

Our study has limitations of the small number of patients included in the cohort and the lack of an invasive reference method to measure GFR, but we are working to expand the number of patients in a larger study to confirm the results.

Overall, our study shows that the criteria proposed by IMWG may underestimate renal disease, particularly in female patients with MM. The equations based on CysC (CKD-EPI-CysC and CAPA) are easier to implement and more sensitive than those based on sCr for the identification of renal impairment in patients with MM. CysC-based equations may also identify patients with MM with more risk factors, which could lead to an early detection and personalised treatment of these patients.

## Figures and Tables

**Table 1:** Characteristics of the cohort of newly diagnosed and untreated multiple myeloma patients enrolled in the study.

Variable	Patients (*n* = 61)
Sex	
Female	24 (39)
Male	37 (61)
Mean age (years)	68 ± 11
Heavy-light chain MM	
IgA-kappa	9 (15%)
IgG-kappa	21 (34%)
IgG-lambda	9 (15%)
IgA-lambda	14 (23%)
Light chain MM	
Kappa	2 (3%)
Lambda	6 (10%)
R-ISS stage at diagnosis	
1	16 (26)
2	31 (51)
3	14 (23)
PET/CT scan (*n* = 50)	
Normal	21 (42)
<5 lesions	11 (22)
5–20 lesions	14 (28)
>20 lesions	4 (8)
Bone marrow plasmacytosis >50%	16 (26)
Adverse cytogenetics (del (17p), t (4,14), t (14,16))	11 (18)
Biochemical phenotype	
Kappa (mg/mL)	25 (12.5–15.1)
Lambda (mg/mL)	24 (10–71)
Kappa/lambda ratio	0.87 (0.12–13.2)
Serum monoclonal component (g/L)	22.5 ± 17
Urine monoclonal component (g/L)	0.08 (0.03–0.18)
Proteinuria (g/L)	0.27 (0.23–0.56)
Albumin (g/L)	35.7 ± 6.3
Hemoglobin (g/dL)	11.4 ± 2.5
Calcium (mg/dL)	10.9 ± 1.1
Phosphate (mg/dL)	4.1 ± 1.3
LDH (U/L)	406 ± 200
*β*-2-Microglobulin (mg/L)	6.5 ± 5.5
Uric acid (mg/dL)	6.5 ± 2.5
Creatinine (mg/dL)	1.35 ± 1.2
Cystatin C (mg/L)	1.67 ± 0.93

**Table 2:** Chronic kidney disease stage classification of patients with newly diagnosed multiple myeloma depending on the equation employed.

Stage	eGFR (mL/min)	CKD-EPI-sCr-CysC (gold standard)	CKD-EPI-sCr	CKD-EPI-CysC	CAPA
G1	≥90	5	16	4	3
G2	89–60	28	24	22	23
G3a	45–59	9	5	7	9
G3b	30–44	4	5	11	11
G4	15–29	13	9	13	12
G5	<15	2	2	4	3
CKD based on KDIGO guidelines (women/men)	<60	28 (10/18)	21 (6/15)	35 (14/21)	35 (14/21)
CKD based on IMWG guidelines (women/men)	<40	17 (4/13)	16 (3/13)	24 (7/17)	23 (6/17)

**Table 3:** Comparison of risk factors associated with reduced glomerular filtration rate estimated by CKD-EPI-creatinine and cystatin C.

	CKD-EPI-CysC		*P* value	CKD-EPI-sCr		*P* value
	eGFR <60	eGFR >60		eGFR <60	eGFR >60	
Number of patients (%)	35 (57)	26 (43)		21 (34)	40 (66)	
Number of women (%)	14 (40)	10 (38)	0.924	6 (28.6)	18 (45)	0.819
Mean age (years ± SD)	71 ± 11	64 ± 10	0.013	72 ± 12	66 ± 9	0.0317
*β*-2-Microglobulin >3.5 mg/L	29 (83)	9 (35)	0.018	20 (95)	18 (45)	0.023
	8.9 ± 6.3	3.38 ± 1.47	0.0001	10.9 ± 6.9	4.2 ± 2.2	<0.0001
Hemoglobin <10 g/dL	14 (40)	4 (15)	0.740	9 (43)	9 (22)	0.769
	10.4 ± 1.9	12.6 ± 2.7	0.0004	10.5 ± 1.97	11.8 ± 2.6	0.0495
Serum urate (mg/dL)	7.1 ± 2.7	5.7 ± 1.81	0.025	8.4 ± 2.5	5.5 ± 1.7	<0.0001
Mean urine monoclonal component (g/L) (95% CI)	0.2 (0.07–0.54)	0.015 (0–0.12)	0.003	0.32 (0.07–0.84)	0.03 (0–0.12)	0.0021
Mean 24 h-urine proteinuria (g/L) (95% CI)	0.53 (0.25–1.29)	0.21 (0.09–0.31)	0.004	1.24 (0.29–1.84)	0.23 (0.11–0.39)	0.0002
Albumin <35 g/L	21 (60)	5 (14)	0.176	9 (43)	19 (47.5)	0.854
	34.1 ± 6.8	38.05 ± 4.77	0.0137	34.8 ± 6.5	36.6 ± 6	0.31
R-ISS-3	13 (37.1)	1 (3.8)	0.002	10 (47.6)	4 (10)	0.003

## Data Availability

The data used to support the findings of this study are included within the article. Other data are restricted in order to protect patient privacy.
